# Solidão e sua associação com indicadores sociodemográficos e de saúde em adultos e idosos brasileiros: ELSI-Brasil

**DOI:** 10.1590/0102-311XPT213222

**Published:** 2023-07-21

**Authors:** Paulo Afonso Sandy, Flávia Silva Arbex Borim, Anita Liberalesso Neri

**Affiliations:** 1 Universidade Estadual de Campinas, Campinas, Brasil.; 2 Faculdade de Ciências Médicas, Universidade Estadual de Campinas, Campinas, Brasil.

**Keywords:** Solidão, Depressão, Autocuidado, Saúde do Idoso, Adulto, Loneliness, Depression, Self Care, Health of the Elderly, Adult, Soledad, Depresión, Autocuidado, Salud del Anciano, Adulto

## Abstract

O objetivo foi investigar a prevalência de solidão e suas associações com indicadores sociodemográficos e de saúde em amostra nacionalmente representativa de adultos e idosos brasileiros. Foram analisados dados da linha de base (2015-2016) do *Estudo Longitudinal da Saúde dos Idosos Brasileiros* (ELSI-Brasil) e incluídos os participantes com informações completas nas variáveis de interesse (n = 7.957). Solidão foi a variável de desfecho, cuja medida baseou-se na pergunta “Com que frequência o(a) senhor(a) se sentiu sozinho(a) ou solitário(a): sempre, algumas vezes ou nunca?”. As variáveis independentes compreenderam indicadores sociodemográficos e comportamentos e condições de saúde. As análises incluíram o teste qui-quadrado de Pearson, para cálculo das frequências relativas, e a regressão de Poisson, para estimativa das razões de prevalência (RP) e respectivos intervalos de 95% de confiança (IC95%). A prevalência de sempre sentir solidão foi de 16,8%; de algumas vezes, 31,7%; e de nunca, 51,5%. Foram observadas associações significativas entre sempre sentir solidão e depressão (RP = 4,49; IC95%: 3,93-5,11), morar só (RP = 2,44; IC95%: 2,12-2,82), baixa escolaridade (RP = 1,93; IC95%: 1,61-2,32), sexo feminino (RP = 1,53; IC95%: 1,36-1,72), autoavaliação de saúde ruim/muito ruim (RP = 1,48; IC95%: 1,27-1,73) e qualidade do sono ruim/muito ruim (RP = 1,21; IC95%: 1,05-1,41). Dado seu potencial de prejuízo à qualidade de vida, é necessário conhecer longitudinalmente as trajetórias da solidão e as variáveis associadas e usar esse conhecimento para o delineamento de políticas públicas e intervenções em saúde que poderão beneficiar o bem-estar biopsicossocial de adultos e idosos brasileiros.

## Abstract (en)

This study aimed to investigate the prevalence of loneliness and its associations with sociodemographic and health indicators in a nationally representative sample of Brazilian adults and older adults. Data from the baseline (2015-2016) of the *Brazilian Longitudinal Study of Aging* (ELSI-Brazil) were analyzed, and participants with complete information on the variables of interest (n = 7,957) were included. Loneliness was the outcome variable, which was based on the question “How often do you feel alone or lonely: always, sometimes, or never?” Independent variables included sociodemographic indicators and health behaviors and conditions. The analyses included the Pearson’s chi-square test for calculating relative frequencies, and Poisson regression for estimating prevalence ratios (PR) and their respective 95% confidence intervals (95%CI). The prevalence of always feeling lonely was 16.8%; sometimes, 31.7%; and never, 51.5%. Significant associations were observed between always feeling lonely and depression (PR = 4.49; 95%CI: 3.93-5.11), living alone (PR = 2.44; 95%CI: 2.12-2.82), low education level (PR = 1.93; 95%CI: 1.61-2.32), being a woman (PR = 1.53; 95%CI: 1.36-1.72), self-rated poor/very poor health (PR = 1.48; 95%CI: 1.27-1.73), and poor/very poor sleep quality (PR = 1.21; 95%CI: 1.05-1.41). Given its potential to harm quality of life, it is necessary to longitudinally understand the trajectories of loneliness and associated variables, and to use this knowledge to design public policies and health interventions that could benefit the biopsychosocial well-being of Brazilian adults and older adults.

## Resumen

El objetivo fue investigar la prevalencia de la soledad y sus asociaciones con indicadores sociodemográficos y de salud en una muestra nacionalmente representativa de adultos y ancianos brasileños. Se analizaron datos de la línea base (2015-2016) del *Estudio Longitudinal de la Salud de los Ancianos Brasileños* (ELSI-Brasil) y se incluyeron los participantes con información completa en las variables de interés (n = 7.957). La soledad fue la variable de desenlace, cuya medida se basó en la pregunta “¿Con qué frecuencia te sentiste solo(a) o solitario(a): siempre, a veces o nunca?”. Las variables independientes incluyeron indicadores sociodemográficos y comportamientos y condiciones de salud. Los análisis incluyeron la prueba de chi-cuadrado de Pearson para calcular las frecuencias relativas, y la regresión de Poisson, para estimar las razones de prevalencia (RP) y los respectivos intervalos de 95% de confianza (IC95%). La prevalencia de sentirse siempre solo fue de 16,8%; a veces, 31,7%; y nunca, 51,5%. Se observaron asociaciones significativas entre sentir siempre soledad y depresión (RP = 4,49; IC95%: 3,93-5,11), vivir solo (RP = 2,44; IC95%: 2,12-2,82), baja escolaridad (RP = 1,93; IC95%: 1,61-2,32), sexo femenino (RP = 1,53; IC95%: 1,36-1,72), autoevaluación de salud mala/muy mala (RP = 1,48; IC95%: 1,27-1,73) y calidad del sueño mala/muy mala (RP = 1,21; IC95%: 1,05-1,41). Dado su potencial para perjudicar la calidad de vida, es necesario conocer longitudinalmente las trayectorias de la soledad y las variables asociadas, y usar ese conocimiento para diseñar políticas públicas e intervenciones en salud que puedan beneficiar el bienestar biopsicosocial de adultos y ancianos brasileños.

## Introdução

A solidão é definida como um sentimento negativo e doloroso ou como a experiência emocional aversiva, individual e privada de que as relações sociais disponíveis são insuficientes para satisfazer as necessidades ou para proporcionar o grau de intimidade emocional desejada pelo indivíduo ^1^^,^^2^. Embora haja um estereótipo muito difundido que associa a solidão à velhice ^3^, ela acontece em todas as faixas etárias ^4^. Entre adultos não institucionalizados de 50 anos ou mais, no período anterior à pandemia de COVID-19, a prevalência de sempre sentir solidão foi de 18% nos Estados Unidos ^5^ e no Reino Unido ^6^. No Brasil, a prevalência variou de 5,1% ^7^ a 14,9% ^8^.

Conceitualmente, a solidão difere do isolamento social, que é definido como um estado objetivo medido via indicadores, tais como viver sozinho, contatos sociais pouco frequentes, baixos níveis de atividade social ou objetiva escassez de parceiros sociais e de interações com outras pessoas ^9^. A solidão pode ser vivenciada independentemente da quantidade de parceiros sociais ou interações que o indivíduo estabelece com eles ^10^, embora, em idosos, ela esteja frequentemente ligada à perda de relações sociais resultante de condições e eventos adversos da vida, de ocorrência comum nessa faixa etária, tais como morte de cônjuge, parentes e amigos e problemas de saúde física e mental que limitam a mobilidade ^11^. No Brasil, apesar de a prevalência de solidão em adultos com 50 anos ou mais residentes na comunidade ter diminuído nos primeiros meses da pandemia de COVID-19, a baixa frequência de contatos sociais continuou significativamente associada a sempre sentir solidão ^7^^,^^12^.

Assim como outros fatores com efeitos já bem estabelecidos, como a obesidade e o tabagismo, a solidão é um fator de risco para mortalidade prematura ^10^. Está associada à autoavaliação de saúde negativa, um indicador confiável do estado de saúde física e preditor robusto de morbimortalidade em idosos ^13^. É bem conhecida a relação entre solidão e problemas de saúde mental, como pior qualidade do sono ^14^ e depressão ^13^. A associação entre solidão e condições de saúde é complexa, mas pode ser explicada por mecanismos fisiológicos e psicológicos ^15^ e pela relação com comportamentos nocivos à saúde ^16^, como sedentarismo ^17^, dietas não saudáveis ^17^, tabagismo ^18^ e etilismo ^16^.

Conhecer a prevalência de solidão em adultos e idosos brasileiros, na presença de variáveis de saúde física e mental e de indicadores sociodemográficos, é uma forma de compreender as interações biopsicossociais que moldam a saúde e o bem-estar dessa população ^19^. Não foram encontradas pesquisas brasileiras de base populacional sobre relações entre níveis de solidão, condições de saúde (qualidade do sono, sintomas depressivos e autoavaliação de saúde), comportamentos de saúde (qualidade da dieta e prática regular de atividade física) e indicadores sociodemográficos (sexo, idade, escolaridade e arranjo de moradia) em adultos mais velhos e idosos. O objetivo deste estudo foi investigar a prevalência de solidão, de acordo com sua intensidade, e suas associações com variáveis sociodemográficas e de saúde em amostra nacionalmente representativa de adultos e idosos brasileiros não institucionalizados com 50 anos ou mais.

## Métodos

Trata-se de pesquisa transversal cujos dados foram extraídos do banco eletrônico da linha de base (2015-2016) do *Estudo Longitudinal da Saúde dos Idosos Brasileiros* (ELSI-Brasil). O ELSI-Brasil é um estudo de base domiciliar, conduzido em amostra nacional representativa da população brasileira não institucionalizada com 50 anos e mais, residente em 70 municípios das cinco regiões macrogeográficas do país. O processo amostral utilizou um delineamento que contemplou a seleção por estágios que combinaram a estratificação de unidades primárias de amostragem (municípios) e, em cada um deles, de setores censitários e domicílios. Todos os moradores dos domicílios selecionados que tivessem 50 anos ou mais foram considerados elegíveis para a entrevista. Mais detalhes podem ser vistos no site da pesquisa (https://elsi.cpqrr.fiocruz.br/) e em outra publicação ^20^.

O banco de dados está localizado em um repositório mantido pela Fundação Oswaldo Cruz (Fiocruz) e disponibilizado para os pesquisadores interessados, mediante senha, no site http://elsi.cpqrr.fiocruz.br/data-access/ (acessado em 01/Ago/2021).

### Participantes

**Figura 1:**
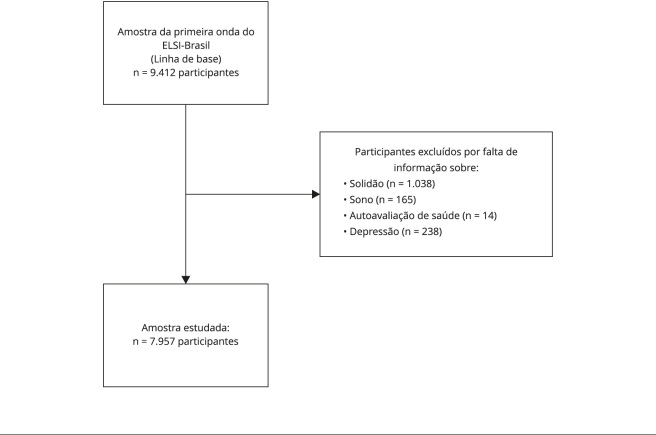
Fluxo das decisões relativas à construção da amostra.

A amostra foi formada por adultos e idosos brasileiros não institucionalizados, com 50 anos ou mais, que tinham registros completos de resposta às variáveis de interesse desta pesquisa e que responderam ao item escalar sobre solidão sem ajuda de um mediador. A Figura 1 informa sobre os passos da construção da amostra, observando esses requisitos.

ELSI-Brasil: *Estudo Longitudinal da Saúde dos Idosos Brasileiros.*

### Variáveis e medidas

Neste estudo, solidão foi a variável dependente. Foram considerados como variáveis independentes os indicadores sociodemográficos sexo, idade, escolaridade e arranjos de moradia, os comportamentos de saúde qualidade da dieta e atividade física e as condições de saúde qualidade do sono, sintomas depressivos e autoavaliação de saúde.

A solidão foi definida como um sentimento negativo e doloroso, ou uma experiência emocional aversiva, individual e privada de que as relações sociais disponíveis são insuficientes para satisfazer as necessidades do indivíduo ou para proporcionar-lhe a intimidade emocional desejada ^1^^,^^2^. Foi avaliada pelo item “Com que frequência o(a) senhor(a) se sentiu sozinho(a) ou solitário(a): nunca, algumas vezes ou sempre?”. Na regressão multivariada, solidão foi dicotomizada em sempre sentir solidão versus algumas vezes/nunca sentir solidão.

Os indicadores sociodemográficos foram investigados por itens de autorrelato por meio dos quais os entrevistados informaram sobre sexo (com as opções masculino e feminino); idade cronológica, posteriormente confirmada pela data do nascimento constante dos registros de cada participante e categorizada em faixas de 50-59, 60-69, 70-79 e 80 anos ou mais; escolaridade (a resposta à pergunta sobre qual foi o último ano em que o(a) participante foi aprovado(a) na escola serviu para calcular o tempo de escolaridade); e arranjo de moradia, em que se perguntou “No total, quantas pessoas moram nesta casa?” (considerando-se como moradores as pessoas que tinham o domicílio como local de residência habitual na data da entrevista), as respostas foram posteriormente codificadas como três ou mais, dois e sozinho(a).

Um dos comportamentos de saúde avaliados foi a qualidade da dieta, referente à frequência do consumo semanal de alimentos que compõem uma dieta saudável ou não saudável, a primeira composta por itens que favorecem a saúde (frutas, hortaliças e carne de frango ou galinha) e a segunda composta por itens que expõem o organismo a riscos à saúde (carne vermelha, de boi, porco ou cabrito). O instrumento que gerou o indicador de qualidade da dieta foi composto por cinco itens com cinco frequências cada um: todos os dias da semana (= 4), em cinco ou seis dias da semana (= 3), em três ou quatro dias da semana (= 2), em um ou dois dias da semana (= 1) e quase nunca ou nunca (= 0). Para os alimentos que se configuram como risco à saúde, a pontuação foi invertida. Os pontos eram somados e o total podia variar de 0 a 16. Quanto maior a pontuação, melhor a qualidade da alimentação. A nova variável foi categorizada considerando-se os tercis da distribuição: dieta de boa qualidade ou alimentação saudável (= melhor tercil), dieta de qualidade intermediária (= tercil intermediário) e dieta de má qualidade ou alimentação não saudável (= pior tercil) ^21^^,^^22^.

Outro comportamento de saúde avaliado foi o nível de prática de atividade física, realizado por meio da versão breve do *Questionário Internacional de Atividade Física* (*International Physical Activity Questionnaire* - IPAQ), traduzido e validado para o Brasil ^23^. Foram consideradas apenas as atividades realizadas por pelo menos 10 minutos contínuos na semana anterior à entrevista. Seguindo a recomendação da Organização Mundial da Saúde (OMS), foram considerados ativos os indivíduos que praticavam atividade física moderada por pelo menos 150 minutos por semana ou aqueles com prática semanal de atividade física vigorosa por pelo menos 75 minutos ^24^.

A qualidade do sono foi avaliada pela pergunta: “Como o senhor avalia a qualidade do seu sono: muito boa, boa, mais ou menos, ruim ou muito ruim?”. As intensidades extremas foram somadas (boa/muito boa = 3 e ruim/muito ruim = 1; mais ou menos = 2). Os sintomas depressivos foram avaliados pela escala CES-D8 (versão de oito itens do *Center for Epidemiological Studies Depression Scale*) ^25^. As respostas afirmativas aos itens que descreviam sintomas depressivos foram somadas. O ponto de corte utilizado foi ≥ 4, a partir do critério adotado por McGovern & Nazroo ^26^, Zaninotto et al. ^27^ e Poole & Jackowska ^28^. A autoavaliação da saúde baseou-se na pergunta “Em geral, como avalia sua saúde: muito boa, boa, regular, ruim ou muito ruim?”. Às intensidades muito boa/boa, regular e ruim/muito ruim foram atribuídos 3, 2 e 1 pontos, respectivamente.

### Análise de dados

Foi realizada análise descritiva das características dos participantes e segundo os níveis de solidão (sempre, algumas vezes ou nunca). As medidas de frequência, seus respectivos intervalos de 95% de confiança (IC95%) e a comparação entre os níveis de solidão foram obtidos pelo teste qui-quadrado de Pearson. As análises das associações entre as variáveis independentes e o desfecho (sempre *versus* algumas vezes/nunca sentir solidão) foram baseadas em razões de prevalência (RP) e IC95%, estimados por meio de análises de regressão de Poisson univariada e multivariada. Foram incluídas no modelo de regressão múltipla as variáveis independentes que, na análise univariada, apresentaram associações com a variável dependente com significância estatística, indicadas por p < 0,20. No modelo final, permaneceram aquelas que exibiram valores de significância, indicadas por p < 0,05. A análise dos dados foi realizada com os comandos *svy* do software Stata, versão 15.0 (https://www.stata.com), utilizando-se as ponderações decorrentes do desenho amostral.

### Cuidados éticos

O ELSI-Brasil foi aprovado pelo Comitê de Ética da Fiocruz, Minas Gerais, e o processo está cadastrado na Plataforma Brasil (CAAE: 34649814.3.0000.5091). Os participantes assinaram termos de consentimento livre e esclarecido para cada um dos procedimentos da pesquisa e autorizaram acesso a bancos de dados secundários correspondentes. O ELSI-Brasil foi financiado pelo Ministério da Saúde: Departamento de Ciência e Tecnologia/Secretaria de Ciência, Tecnologia e Insumos Estratégicos (DECIT/SCTIE, Processos: 404965/2012-1 e TED 28/2017); Coordenação de Saúde da Pessoa Idosa na Atenção Primária/Departamento de Gestão do Cuidado Integral/Secretaria de Atenção Primária à Saúde (COPID/DECIV/SAPS, Processos: 20836, 22566, 23700, 25560, 25552 e 27510).

## Resultados

**Figura 2:**
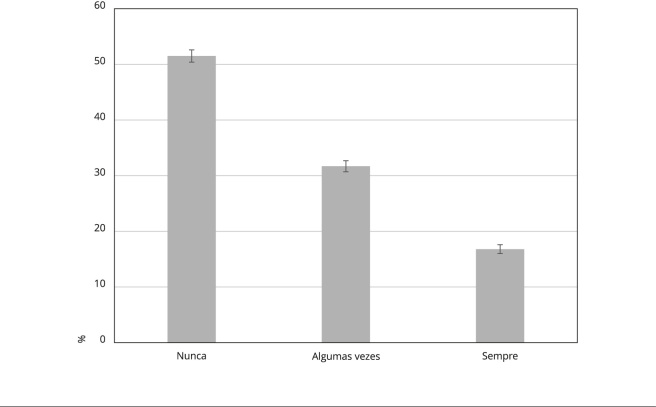
Prevalência de solidão por níveis de intensidade entre brasileiros com 50 anos ou mais residentes na comunidade (n = 7.957). *Estudo Longitudinal da Saúde dos Idosos Brasileiros* (ELSI-Brasil), 2015-2016.

A prevalência de sempre sentir solidão foi de 16,8%; a de algumas vezes sentir solidão, 31,7%; e a de nunca sentir solidão, 51,5% (Figura 2). A maioria dos participantes eram mulheres, tinham 60 anos ou mais, baixa (quatro anos ou menos) ou nenhuma escolaridade formal; residiam em arranjos domiciliares múltiplos (com três ou mais moradores); tinham dieta classificada como saudável ou de qualidade intermediária; eram fisicamente inativos; avaliaram a qualidade do sono como boa/muito boa; não tinham sintomas sugestivos de depressão e avaliaram a própria saúde como boa/muito boa ou de qualidade intermediária. A resposta “sempre senti solidão” foi significativamente mais frequente entre mulheres, participantes de 80 anos ou mais, sem escolaridade formal, pessoas que moravam sozinhas, com alimentação não saudável, com sono ruim/muito ruim, com pontuação superior à nota de corte para depressão e com autoavaliação de saúde ruim/muito ruim (Tabela 1).

**Tabela 1:** Características dos participantes do estudo, total e segundo os níveis de solidão. *Estudo Longitudinal da Saúde dos Idosos Brasileiros* (ELSI-Brasil), 2015-2016.

	% (IC95%)	Sentimento de solidão		
		Nunca	Algumas vezes	Sempre
Sexo			p < 0,001 *	
Masculino	44,0 (43,0-45,1)	63,3	27,5	9,2
Feminino	56,0 (54,8-57,0)	44,1	36,2	19,7
Idade (anos)			p < 0,001 *	
50-59	45,3 (44,2-46,4)	50,3	35,8	13,9
60-69	31,4 (30,3-32,4)	55,2	29,6	15,2
70-79	17,6 (16,8-18,5)	58,2	26,0	15,8
80 ou mais	5,7 (5,1-6,2)	53,0	28,3	18,7
Escolaridade (anos de estudo)			p < 0,001 *	
9 ou mais	27,0 (26,0-28,0)	53,2	37,8	9,0
5-8	20,7 (19,8-21,6)	52,8	35,1	12,1
1-4	38,3 (37,2-39,4)	53,2	29,1	17,7
Nunca foram à escola	14,0 (13,2-14,7)	52,8	21,5	25,7
Arranjos de moradia			p < 0,001 *	
Com 3 pessoas ou mais	56,0 (54,9-57,1)	55,3	32,5	12,2
Com 2 pessoas	31,4 (30,4-32,4)	54,3	31,1	14,6
Moram sozinhos	12,6 (11,8-13,3)	33,7	33,8	32,5
Qualidade da dieta			p = 0,001 *	
Saudável ou boa	31,4 (30,3-32,4)	54,0	32,2	13,8
Intermediária	35,2 (34,2-36,3)	53,5	33,5	13,0
Não saudável ou ruim	33,4 (32,3-34,4)	51,6	30,7	17,7
Atividade física			p = 0,176	
Ativos	26,2 (25,3-27,2)	51,1	33,7	15,2
Inativos	73,8 (72,8-74,7)	53,7	31,6	14,6
Qualidade do sono			p < 0,001 *	
Boa e muito boa	54,2 (53,0-55,2)	61,8	27,7	10,5
Intermediária	27,3 (26,4-28,3)	46,4	37,9	15,7
Ruim e muito ruim	18,5 (17,6-19,3)	36,6	36,8	26,6
Sintomas depressivos			p < 0,001 *	
Não (CES-D8 < 4)	65,2 (64,1-66,2)	68,9	25,5	5,6
Sim (CES-D8 ≥ 4)	34,8 (33,8-35,9)	21,8	45,3	32,9
Autoavaliação de saúde			p < 0,001 *	
Boa/Muito boa	43,1 (42,0-44,2)	61,1	29,1	9,8
Intermediária	45,3 (44,2-46,3)	49,4	35,2	15,4
Ruim/Muito ruim	11,6 (10,9-12,3)	34,9	32,1	33,0

CES-D8: versão de oito itens do *Center for Epidemiological Studies Depression Scale*; IC95%: intervalo de 95% de confiança.

* Diferenças estatisticamente significativas para p < 0,05, teste qui-quadrado de Pearson.

Na análise múltipla (Tabela 2), a solidão mostrou-se positivamente associada a: sexo feminino (RP = 1,53; IC95%: 1,36-1,72); escolaridade até quatro anos (RP = 1,47; IC95%: 1,22-1,77 para indivíduos com um a quatro anos de estudo e RP = 1,93; IC95%: 1,61-2,32 para aqueles que nunca foram à escola); arranjos de moradia com duas pessoas (RP = 1,20; IC95%: 1,05-1,37) ou uma (RP = 2,44; IC95%: 2,12-2,82); qualidade do sono ruim/muito ruim (RP = 1,21; IC95%: 1,05-1,41); sintomas depressivos (RP = 4,49; IC95%: 3,93-5,11) e autoavaliação de saúde ruim/muito ruim (RP = 1,48; IC95%: 1,27-1,73).

**Tabela 2:** Razões de prevalência (RP) brutas e ajustadas de solidão, considerando indicadores sociodemográficos, comportamentos em saúde e condições de saúde. *Estudo Longitudinal da Saúde dos Idosos Brasileiros* (ELSI-Brasil), 2015-2016.

	RP bruta (IC95%)	RP ajustada * (IC95%)
Sexo		
Masculino	1,00	1,00
Feminino	2,15 (1,88-2,46)	1,53 (1,36-1,72)
Idade (anos)		
50-59	1,00	
60-69	1,09 (0,94-1,26)	
70-79	1,13 (0,94-1,37)	
80 ou mais	1,34 (1,03-1,75)	
Escolaridade (anos de estudo)		
9 ou mais	1,00	1,00
5-8	1,34 (1,05-1,71)	1,09 (0,87-1,36)
1-4	1,96 (1,61-2,39)	1,47 (1,22-1,77)
Nunca foram à escola	2,85 (2,34-3,48)	1,93 (1,61-2,32)
Arranjos de moradia		
Com 3 pessoas ou mais	1,00	1,00
Com 2 pessoas	1,20 (1,02-1,40)	1,20 (1,05-1,37)
Moram sozinhos	2,66 (2,30-3,08)	2,44 (2,12-2,82)
Qualidade da dieta		
Saudável ou boa	1,00	
Intermediária	0,94 (0,82-1,09)	
Não saudável ou ruim	1,28 (1,08-1,52)	
Atividade física		
Ativos	1,00	
Inativos	0,96 (0,84-1,08)	
Qualidade do sono		
Boa/Muito boa	1,00	1,00
Intermediária	1,50 (1,27-1,76)	1,10 (0,96-1,27)
Ruim/Muito ruim	2,54 (2,11-3,06)	1,21 (1,05-1,41)
Sintomas depressivos		
Não (CES-D8 < 4)	1,00	1,00
Sim (CES-D8 ≥ 4)	5,85 (5,04-6,78)	4,49 (3,93-5,11)
Autoavaliação de saúde		
Boa/Muito boa	1,00	1,00
Intermediária	1,57 (1,32-1,85)	1,10 (0,95-1,26)
Ruim/Muito ruim	3,37 (2,79-4,06)	1,48 (1,27-1,73)

CES-D8: versão de oito itens do *Center for Epidemiological Studies Depression Scale*; IC95%: intervalo de 95% de confiança.

* Modelo ajustado pelas variáveis que apresentaram p < 0,20 no teste que avaliou associações entre as variáveis independentes e solidão.

## Discussão

Este estudo avaliou a prevalência de solidão de acordo com seus níveis de intensidade em adultos brasileiros não institucionalizados com 50 anos ou mais, antes da pandemia de COVID-19, e suas associações com variáveis sociodemográficas e comportamentos e condições de saúde. Embora a maior parte dos participantes tenha declarado nunca se sentir solitária, foi observada maior probabilidade de sempre sentir solidão entre mulheres, pessoas que moravam sozinhas, que nunca foram à escola, que pontuaram para depressão e as que avaliaram a própria saúde e a qualidade do sono como ruins/muito ruins.

Os achados deste estudo mostram que o sentimento de solidão intensa é mais frequente entre idosos de 80 anos ou mais, quando comparados aos idosos mais jovens. Esses resultados são consistentes com os de estudos prévios, ainda que o sentimento de solidão nem sempre seja mais prevalente em idosos com 80 anos ou mais em comparação a adultos mais jovens ^29^^,^^30^. Contudo, essa relação não manteve a significância após ajuste para variáveis sociodemográficas e condições de saúde. Na literatura, não existe consenso quanto à distribuição e à intensidade da solidão em relação às idades ^4^^,^^13^^,^^31^. A idade pode não ser um fator de risco per se para solidão, mas sim um construto mais amplo, que reflita experiências de vida (como a viuvez e a aposentadoria), recursos materiais e condição de saúde. Ou seja, o idoso muito idoso teria mais dificuldade em alcançar de forma satisfatória suas expectativas em relação às suas relações interpessoais e em compensar o sentimento de solidão por apresentar mais limitações físicas e por vivenciar mais perdas sociais que os adultos mais jovens, dificultando seu engajamento em novas atividades e a construção de novas relações ^4^^,^^31^.

O dado que revela a maior intensidade de solidão entre mulheres do que entre homens é concordante com os achados prévios de que os níveis de solidão em mulheres idosas são mais altos que os dos homens quando se usa instrumento direto de medida de solidão ^29^. Entretanto, a relação entre solidão e sexo ainda é incerta e pode estar relacionada, entre outros fatores, ao tipo de instrumento usado para medi-la (isto é, se a medida é direta ou indireta) ^32^. Acredita-se que, por razões culturais, os homens tendem a esconder seus sentimentos e a emitir respostas socialmente mais desejáveis, uma vez que ser considerado solitário tem uma conotação social negativa e estigmatizante ^33^. Também é possível que o nível mais alto de solidão observado entre as mulheres seja em parte explicado pelo fato de elas viverem mais do que os homens e de, na idade avançada, estarem mais sujeitas à viuvez e a morar sozinhas do que eles ^13^^,^^34^. Além disso, outra possível explicação seria a maior prevalência de doenças mentais entre as mulheres que entre os homens ^4^, uma associação não explorada pelos autores.

A observação de que houve mais idosos de baixa escolaridade entre os que relataram sempre sentir solidão condiz com o resultado de outro estudo em que essa relação foi analisada ^30^, mas não com achados de metanálise de estudos longitudinais sobre o tema ^13^. É possível que a associação entre solidão e baixa escolaridade reflita uma ligação indireta ou mediada por fatores de risco, entre eles menor renda, mais estresse crônico, além de menor variedade e pior qualidade das relações sociais ^35^. Ao contrário, indivíduos mais escolarizados tendem a angariar mais recursos econômicos, culturais, sociais e intelectuais ao longo da vida, que lhes propiciarão mais condições para enfrentar a solidão na velhice, principalmente diante de adversidades e necessidade de apoio ^30^.

Na pesquisa ora relatada, observou-se que os indivíduos que moravam sozinhos apresentaram níveis mais altos de solidão do que os que moravam com uma ou mais pessoas. Esses achados são congruentes com os do estudo de Hutten et al. ^4^, que mostrou que viver sozinho foi o segundo fator de risco mais importante para solidão em pessoas de 65 anos ou mais, ficando atrás apenas da depressão e da ansiedade. De acordo com a perspectiva de desenvolvimento ao longo de toda a vida (*lifespan*), os fatores de risco para solidão são diferentes para cada estágio da vida, devido à influência de grandes eventos marcadores da experiência de cada faixa etária. Assim, na velhice, torna-se mais provável que viver sozinho reflita o estado de viuvez ^4^. Porém, mais do que viver sozinho em si, a ausência do cônjuge parece ser o que mais torna os idosos vulneráveis ao sentimento de solidão ^36^, especialmente quando são homens ^37^.

Apesar da corresidência com filhos, parentes e amigos não ser um fator de proteção para solidão tão forte quanto viver com um cônjuge ^36^, conviver com outras pessoas pode significar maior suporte emocional e mais oportunidades para socialização. Embora a solidão e o isolamento social sejam construtos distintos, eles se correlacionam ^38^^,^^39^ - a baixa frequência de contatos sociais é um dos mais importantes fatores de risco para solidão em idosos ^4^. Criar e manter contatos sociais pode ser mais difícil para o idoso que mora sozinho, porque requer, entre outras coisas, capacidade física para sair de casa, que pode ser prejudicada pelo aparecimento e agravamento de multimorbidades ^4^.

Neste estudo, contrariando nossas hipóteses, sempre sentir solidão não se relacionou significativamente com comportamentos de saúde (dieta saudável e prática de exercícios físicos). Uma das razões pelas quais se acredita que indivíduos solitários sejam levados ao desengajamento de atividades promotoras de saúde é a relação entre solidão e redução do funcionamento cognitivo, de modo especial as funções executivas, que englobam a capacidade de planejar e tomar decisões, tornando mais difícil a adoção de um estilo de vida saudável ^40^. Por outro lado, mesmo que solitários, os indivíduos podem se envolver com atividades saudáveis em decorrência do estímulo e da pressão de familiares e amigos, pela necessidade de se adequar a normas sociais e devido à oferta de informações sobre promoção de saúde disponíveis nesses ambientes ^41^^,^^42^. É de se ressaltar que a evidência ligando solidão a comportamentos em saúde ainda é controversa e bem mais escassa do que aquelas sobre os efeitos do isolamento social. Há estudos que evidenciam relações entre solidão e baixa qualidade da dieta ^43^, inatividade física ^44^ e tabagismo ^44^ e outros que mostram ausência de conexão entre esses aspectos ^17^^,^^45^.

Os resultados que indicaram associação entre solidão mais frequente e pior autoavaliação de saúde replicam dados obtidos em estudos prévios ^30^^,^^46^^,^^47^. A autoavaliação de saúde é um indicador que pode representar condições de saúde que não são biomedicamente identificáveis, nem são incluídas em um exame médico de rotina, como é o caso da solidão ^48^. Holtfreter et al. ^49^ apontaram que a relação entre solidão e autoavaliação negativa de saúde pode ser reduzida diante da presença de um bom relacionamento com o cônjuge, reforçando a importância da manutenção de relações positivas e de qualidade nessa faixa etária.

Há evidências de que a relação entre autoavaliação negativa de saúde e solidão em idosos possa ser parcialmente mediada por sono de má qualidade ^50^. A associação entre solidão e pior qualidade do sono observada em nosso estudo replica evidências de que adultos e idosos solitários são mais vulneráveis à maior fragmentação do sono, à insônia, à inatividade diurna, à redução do tempo total do sono e ao uso de medicamentos para dormir ^50^. É possível que essa relação seja bidirecional ^14^, mas ainda não se sabe se a redução da qualidade do sono em idosos solitários pode ser parcialmente explicada por nível mais alto de estresse percebido na presença da solidão ^51^ ou se noites de sono ruim reduzem o funcionamento cognitivo e social e prejudicam as relações sociais durante o dia ^52^.

Os resultados reforçam a bem documentada correlação entre solidão e depressão em idosos e adultos na meia-idade. Embora haja evidências de que solidão e depressão são construtos distintos, sabe-se que compartilham sentimentos, tais como percepção negativa das interações sociais, senso antecipado de rejeição, redução das respostas a estímulos positivos e aumento do estresse ^51^. Ainda não está bem estabelecido se a solidão causa depressão ou vice-versa ^53^^,^^54^. Estudos longitudinais com idosos mostraram que o sentimento de solidão em linha de base associou-se ao aparecimento de sintomas depressivos no seguimento ^55^^,^^56^, assim como sintomas depressivos prenunciaram o aparecimento de solidão ^57^.

Este estudo tem limitações. Devido ao seu delineamento transversal, não se pode estabelecer relação temporal nem causal entre as variáveis. A amostra limitou-se a adultos brasileiros não institucionalizados com mais de 50 anos, motivo pelo qual os resultados não podem ser generalizados para a população geral. Foi utilizado instrumento de medida direta e de item único, o que pode ter contribuído para uma subestimativa da prevalência de solidão, especialmente em homens. Os comportamentos e as condições de saúde foram baseados em autorrelatos dos entrevistados, que podem ter sido influenciados pela cognição e pela desejabilidade social. Outras variáveis potencialmente importantes não foram incluídas, entre elas, por exemplo, renda, traços de personalidade, multimorbidade e fragilidade. Todavia, até onde sabemos, este é o primeiro estudo que investiga a prevalência de níveis de solidão em amostra populacional representativa de adultos brasileiros com 50 anos ou mais e suas associações com comportamentos e condições de saúde.

Em conclusão, apesar de neste estudo a maioria de adultos com 50 anos ou mais ter declarado nunca sentir solidão, esse sentimento foi mais intenso entre aqueles com depressão, do sexo feminino, que não frequentaram a escola, que moravam sós, que tinham pior qualidade do sono e que apresentavam percepção negativa da própria saúde. Os dados apontam a solidão como uma condição a ser levada em conta por serviços e profissionais responsáveis pelo cuidado de adultos e idosos, dado seu potencial para prejudicar a qualidade de vida nesses grupos.

São necessários estudos que promovam o conhecimento longitudinal das trajetórias da solidão e suas variáveis associadas. Esse conhecimento poderá servir de base para o delineamento de políticas públicas e ações que poderão ajudar indivíduos mais velhos e solitários a se manterem saudáveis, especialmente diante de doenças que acarretem restrições sociais. No longo prazo, ações governamentais promotoras da igualdade de oportunidades ao longo de toda a vida, além do uso e da avaliação constante de estratégias de intervenção em saúde psicossocial, poderão beneficiar o bem-estar de adultos e idosos brasileiros.
